# Persimmon-derived tannin ameliorates the pathogenesis of ulcerative colitis in a murine model through inhibition of the inflammatory response and alteration of microbiota

**DOI:** 10.1038/s41598-021-86608-1

**Published:** 2021-03-31

**Authors:** Masahiro Kitabatake, Yoko Matsumura, Noriko Ouji-Sageshima, Tatsuki Nishioka, Atsushi Hara, Shin-ichi Kayano, Toshihiro Ito

**Affiliations:** 1 Department of Immunology, Nara Medical University, 840 Shijo-cho, Kashihara, Nara 634-8521 Japan; 2 Department of Nutrition, Faculty of Health Science, Kio University, Kitakatsuragi-gun, 635-0832 Japan

**Keywords:** Ulcerative colitis, Ulcerative colitis, Metagenomics, Dysbiosis, Ulcerative colitis

## Abstract

Ulcerative colitis (UC) is a chronic inflammatory bowel disease (IBD) induced by dysregulation of the immune response in the intestinal mucosa. Although the underlying mechanisms of UC development are not fully understood, disruption of gut microbiota, “dysbiosis”, is thought to lead to the development of IBD. Persimmon (Ebenaceae *Diospyros kaki* Thunb.)-derived tannin, which is a condensed polymeric tannin consisting of catechin groups, has antioxidant, anti-inflammatory, and antimicrobial activities. In this study, we assessed the effect of persimmon-derived tannin on a murine model of UC established by dextran sulfate sodium-induced colitis in female mice. Dietary supplementation of tannin significantly decreased disease activity and colon inflammation. A hydrolysate of tannin directly suppressed expression of inflammatory genes in macrophages in vitro. In faecal microbiota, the relative abundance of *Bacteroides* was increased significantly by tannin supplementation. Alpha-diversity indices in colitis-induced mice were significantly higher in the tannin diet group compared with the control diet group. Additionally, expansion of *Enterobacteriaceae* and *Enterococcus*, which is associated with disease progression of IBD, was remarkably suppressed in the tannin diet group. These results suggest that persimmon-derived tannin ameliorates colon inflammation in UC through alteration of the microbiota composition and immune response, which may be a promising candidate for IBD therapy.

## Introduction

Inflammatory bowel disease (IBD) characterised by chronic relapsing intestinal inflammation has increased in recent years worldwide^1,2^. IBD is mainly divided into two conditions, ulcerative colitis (UC) and Crohn’s disease (CD). Although the etiology of IBD has not been fully elucidated, recent research has indicated that the individual’s genetic susceptibility, external environment, intestinal microbial flora, and immune responses are involved and functionally integrated in the pathogenesis of IBD^3,4^.

Using next-generation sequencing and computational methods to analyse highly complex biological sequence data, the importance of the gut microbiome in IBD has been intensely researched and well documented^5–7^. Many studies have characterised the IBD gut microbiota and its reduced bacterial diversity and richness compared with healthy controls. The imbalance of the commensal bacterial community, dysbiosis of microbiota, contributes to the pathogenesis of IBD. Among the many factors that induce IBD, diet is one of the major factors that influence intestinal microbiota, and several studies have examined the role of specific nutrients in IBD development and the disease course^8–11^. Additionally, pharmacological therapies for IBD management rely on anti-inflammatory drugs, such as non-steroidal anti-inflammatory drugs and anti-tumour necrosis factor (TNF) monoclonal antibodies, which have serious side effects such as secondary infections and immunosuppression. Thus, alternative approaches are required to provide long-term disease management with a low risk of side effects.

Tannins are naturally occurring astringent consisting of a mixture of polyphenols. Various kinds of plants, such as green tea, grape, and persimmon, contain large quantities of tannins^12^. Persimmon (*Diospyros kaki*)-derived tannin is a condensed polymeric tannin consisting of flavan-3-ols such as catechin groups. The major four types of catechins, epicatechin, epicatechin gallate, epigallocatechin, and epigallocatechin gallate, condense at 19–47 molecules and form a large molecule^13^. Polyphenols, including persimmon-derived tannin, have an obvious antioxidant activity in vitro and in vivo, which suppresses oxidative stress caused by reactive oxygen species^14,15^. Additionally, polyphenols have an anti-inflammatory activity. Thus, several studies have reported that polyphenols prevent cancer, cardiovascular diseases, and diabetes. Tannins also have immunomodulatory and antimicrobial activities^12^. We have previously demonstrated that colonic fermentation of persimmon-derived tannin enhances its in vitro antioxidant activity, and a hydrolysate of tannin has antibacterial and anti-inflammatory effects against *Mycobacterium avium* complex (MAC)^15,16^. We also showed that dietary supplementation of a soluble fraction of persimmon-derived tannin attenuates pulmonary MAC infection^16^, which suggests that persimmon-derived tannin affects inflammation in mucosal immunity including the gut through its anti-inflammatory effect and alteration of the gut microbial composition.

In the present study, we evaluated the effect of persimmon-derived tannin on a murine IBD model and compared IBD pathogenesis and bacterial organisms in the faecal microbiota of persimmon-derived tannin and control diet groups.

## Results

### Dietary supplementation of persimmon-derived tannin ameliorates dextran sulfate sodium (DSS)-induced colitis

**Figure 1:**
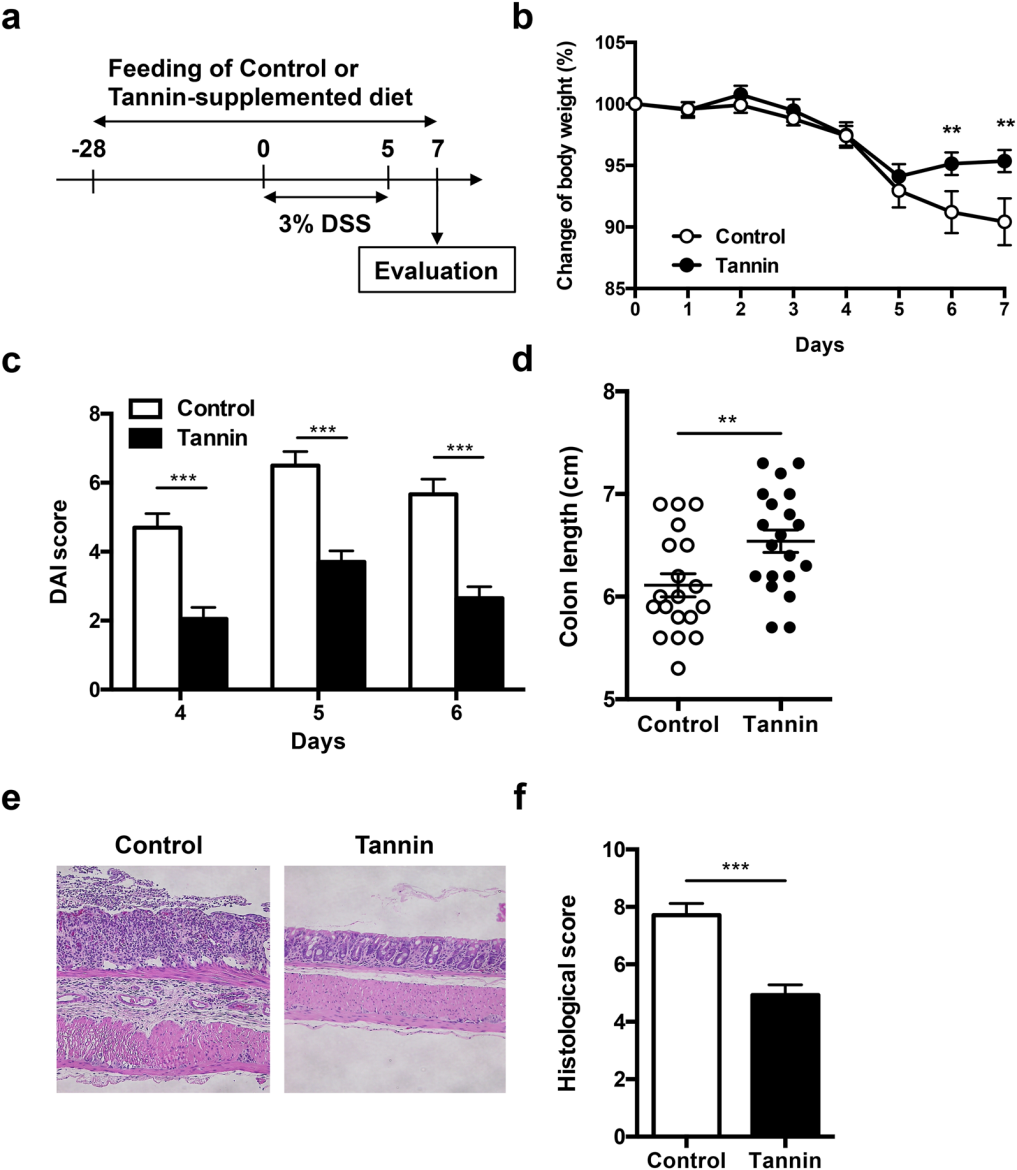
Amelioration of DSS-induced colitis by dietary supplementation of persimmon-derived tannin. (**a**) Schematic model of the experimental design. Female BALB/c mice were fed the control or tannin-supplemented diet for 4 weeks and then administered 3% DSS in drinking water for 5 days. (**b**,**c**) Weight changes (**b**) and disease activity index (**c**) calculated from weight loss, stool consistency, and rectal bleeding were monitored after DSS administration. (**d**) Colon length was measured in colitis-induced mice at day 7. (**e**) Sections of the proximal colon region were stained with haematoxylin and eosin. (**f**) Histological score was calculated from the severity of inflammation, thickness of inflammatory involvement, epithelial damage, and the extent of lesions. Data are shown as the mean ± SEM of three independent experiments (control diet group n = 19, tannin diet group n = 20). **p < 0.01, ***p < 0.001.

We first examined the effect of persimmon-derived tannin on the ulcerative colitis model. Administration of DSS increases intestinal permeability and decreases the barrier function, which may allow invasion of bacteria into the intestinal epithelium to induce robust inflammation^17^. Female mice were fed the control AIN-G93 diet or persimmon-derived tannin-supplemented diet for 4 weeks and then administered 3% DSS in drinking water from day 0 to 5 to induce colitis (total n = 20 in each group) (Fig. 1a). DSS-treated mice fed the control diet manifested visible bloody stool and diarrhoea with weight loss from day 3. One mouse fed the control diet died with severe weight loss at day 6. Weight loss at days 6 and 7 was suppressed in the tannin diet group compared with the control diet group (Fig. 1b). The score of the disease activity index (DAI) evaluated by weight loss, stool consistency, and rectal bleeding was also decreased in the tannin diet group (Fig. 1c). At day 7, the colon length, which was shortening by DSS treatment, was elongated in the tannin diet group (Fig. 1d). Histological analysis by haematoxylin and eosin staining revealed that distortion of the crypt architecture, hyperplasia, and infiltration of leucocytes in the control diet group (Fig. 1e). In contrast, such histological destruction and inflammation were dramatically reduced in the tannin diet group. The histological score assessed by inflammation severity, the thickness of inflammatory involvement, epithelial damage, and the extent of lesions, was lower in the tannin diet group (Fig. 1f). These results indicate that dietary supplementation of persimmon-derived tannin suppresses the inflammation in DSS-induced colitis.

### Effect of persimmon-derived tannin on expression of inflammatory cytokines in the colon

**Figure 2:**
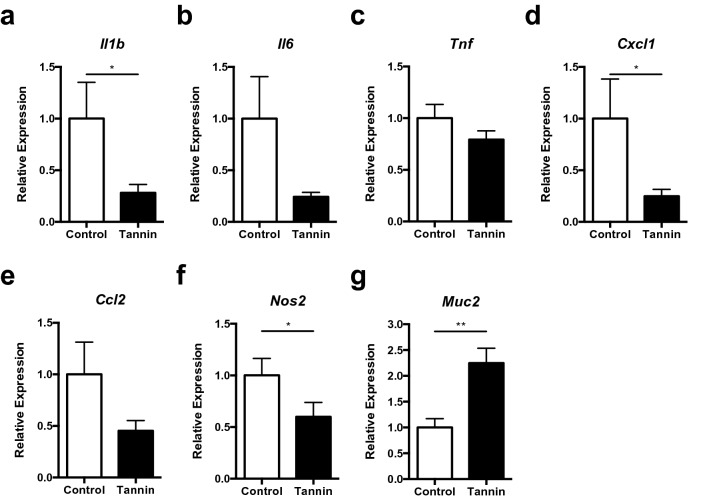
Effect of the tannin-supplemented diet on gene expression in the colon of colitis-induced mice. Transcripts of IL-1β (**a**), IL-6 (**b**), TNF-α (**c**), CXCL1 (**d**), CCL2 (**e**), iNOS (**f**), and Muc2 (**g**) in the colon of colitis-induced mice fed the control or tannin diet were measured by quantitative PCR (normalized with *Gapdh*). Data are shown as the mean ± SEM of three independent experiments (n = 19–20). *p < 0.05, **p < 0.01.

Next, we compared the expression profiles of inflammatory-related genes normalized with *Gapdh* in the DSS-induced colons between control and tannin diet groups. Expression of inflammatory cytokine *Il1b* and chemokine *Cxcl1* was significantly decreased in the tannin diet group (Fig. 2a–e). Expression of *Il6* and *Ccl2* was not changed significantly, but tended to be reduced in the tannin diet group, while expression of *Tnf* was comparable between the two groups. Transcripts of the inducible nitric oxide synthase (iNOS) gene (*Nos2*), a critical enzyme for initiation and maintenance of inflammation in IBD, were also decreased in the tannin diet group (Fig. 2f). In contrast, *Muc2*, which is a major component of inner and outer mucosal layers of the colon, was highly expressed in the tannin diet group (Fig. 2g). We further validated these expression changes normalized with *Tbp* because expression of *Gapdh* was likely up-regulated in inflamed colon^18,19^. Although expression of *Gapdh* normalized with *Tbp* in control diet group was higher than that of tannin diet group, similar results with Fig. 2 were observed by normalization with *Tbp* (Supplementary Fig. S1). These results suggest that persimmon-derived tannin suppresses inflammation and maintains mucus production in the colitis-induced colon.

### Persimmon-derived tannin does not affect the induction of effector T cells in DSS-induced colitis

Previous studies have been reported that various kinds of effector T cells contribute to the pathogenesis of IBD^3^. Therefore, we investigated the influence of dietary supplementation of persimmon-derived tannin on effector T cells in mesenteric lymph nodes and the colon lamina propria. The proportions of effector CD4^+^ T cells, including type 1 helper (Th1; IFN-γ^+^ CD4 T) and type 17 helper (Th17; IL-17A^+^ CD4 T) cells, in mesenteric lymph nodes were not changed significantly in the tannin diet group compared with the control (Supplementary Fig. S2a). Similarly, the proportions of effector T cells in the colon lamina propria were not altered in the tannin diet group (Supplementary Fig. S2b). Consistent with these results, expression of *Ifng* and *Il17a* in the colon was not reduced significantly in the tannin diet group (Supplementary Fig. S2c). Proportions of regulatory T cells (Foxp3^+^ CD4 T) in mesenteric lymph nodes and lamina propria of the tannin diet group were comparable with those of the control diet group (Supplementary Fig. S2a, b). These results suggest that persimmon-derived tannin affects other factors, such as the innate immune response or microbiota, rather than effector T cells.

### Hydrolysate of persimmon-derived tannin suppresses macrophage activation

**Figure 3:**
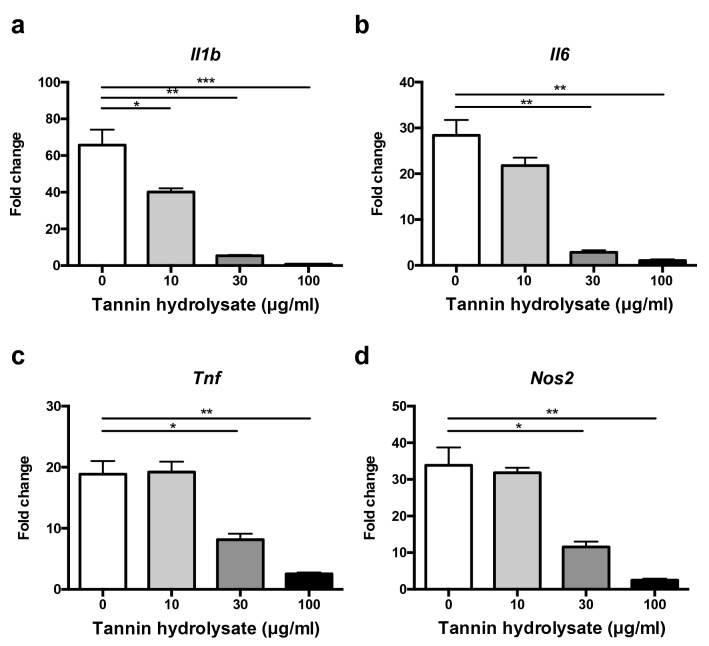
Hydrolysate of tannin suppresses induction of inflammatory genes in stimulated macrophages in vitro. BMMCs were pretreated with tannin hydrolysate at 0–100 µg/ml for 2 h and then stimulated with TLR9 ligand CpG DNA for 4 h. Expression of inflammatory genes IL-1β (**a**), IL-6 (**b**), TNF-α (**c**), and iNOS (d) was measured by quantitative PCR. Data are shown as the mean ± SEM (n = 3). *p < 0.05, **p < 0.01, ***p < 0.001 compared with no treatment with tannin hydrolysate (0 µg/ml).

We previously showed that hydrolysis is required for persimmon-derived tannin to exert its antioxidant and bacteriostatic effects^16^. Therefore, a hydrolysate of tannin was used for its in vitro anti-inflammatory effect against bacterial components. Bone marrow-derived macrophages (BMMCs) were stimulated with CpG, a ligand of Toll-like receptor (TLR) 9, after pretreatment with various concentrations of tannin hydrolysate. Induction of inflammatory cytokines, including *Il1b*, *Il6*, and *Tnf*, and *Nos2* in stimulated macrophages was significantly decreased by pretreatment with tannin hydrolysate in a dose-dependent manner (Fig. 3). This result suggests that dietary supplementation of persimmon-derived tannin attenuates colitis in part through inhibition of macrophage activation.

### Dietary supplementation of persimmon-derived tannin alters the intestinal microbiota composition

**Figure 4:**
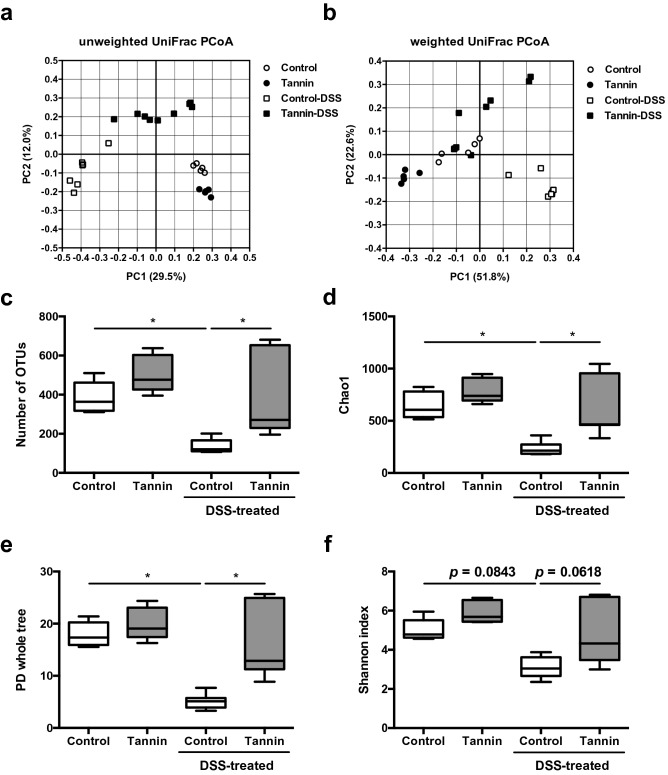
Dietary supplementation of persimmon-derived tannin affects the composition and diversity of microbiota in colitis-induced mice. Compositions of faecal bacteria in untreated and DSS-treated mice fed the control or tannin-supplemented diet were analysed by 16S ribosomal RNA gene sequencing. (**a**, **b**) PCoA of unweighted (**a**) and weighted (**b**) UniFrac distances were plotted. (**c**–**f**) Alpha-diversity indices, number of OTUs (**c**), Chao1 (**d**), PD whole tree (**e**), and Shannon index (**f**) were calculated by QIIME software. Data are shown as the mean ± SEM with individual plots. Untreated mice fed the control diet (n = 5) or tannin diet (n = 5); DSS-treated mice fed the control diet (n = 7) or tannin diet (n = 9). *p < 0.05.

Next, we assessed the effect of dietary supplementation of persimmon-derived tannin on the faecal microbiota. Faeces were collected from untreated and DSS-treated mice fed the control or tannin diet and then 16S ribosomal RNA gene sequencing was performed. Both unweighted and weighted UniFrac-based principal coordinates analysis (PCoA) showed that each group formed a distinct cluster of bacterial composition, which indicated that dietary supplementation of tannin directly altered the microbiota composition and DSS-mediated changes of microbiota were affected by persimmon-derived tannin (Fig. 4a,b). In comparison of control and tannin diets in untreated mice, the indices of α-diversity of microbiota, number of operation taxonomic units (OTUs), Chao1, phylogenetic diversity (PD) whole tree, and Shannon index, tended to increase in the tannin diet group (Fig. 4c–f). Treatment with DSS markedly decreased number of OTUs, Chao1, and PD whole tree in mice fed the control diet. In contrast, these indices in the tannin diet group were not significantly changed by DSS treatment and significantly higher in DSS-treated mice fed the tannin-supplemented diet compared with those fed the control diet. Similar tendency was observed in Shannon index. These results suggest that dietary supplementation of tannin maintains the diversity and richness of bacterial species in the microbiota of colitis-induced mice.

### Dietary supplementation of persimmon-derived tannin suppresses expansion of *Enterobacteriaceae* and *Enterococcus* in the colitis model

**Figure 5:**
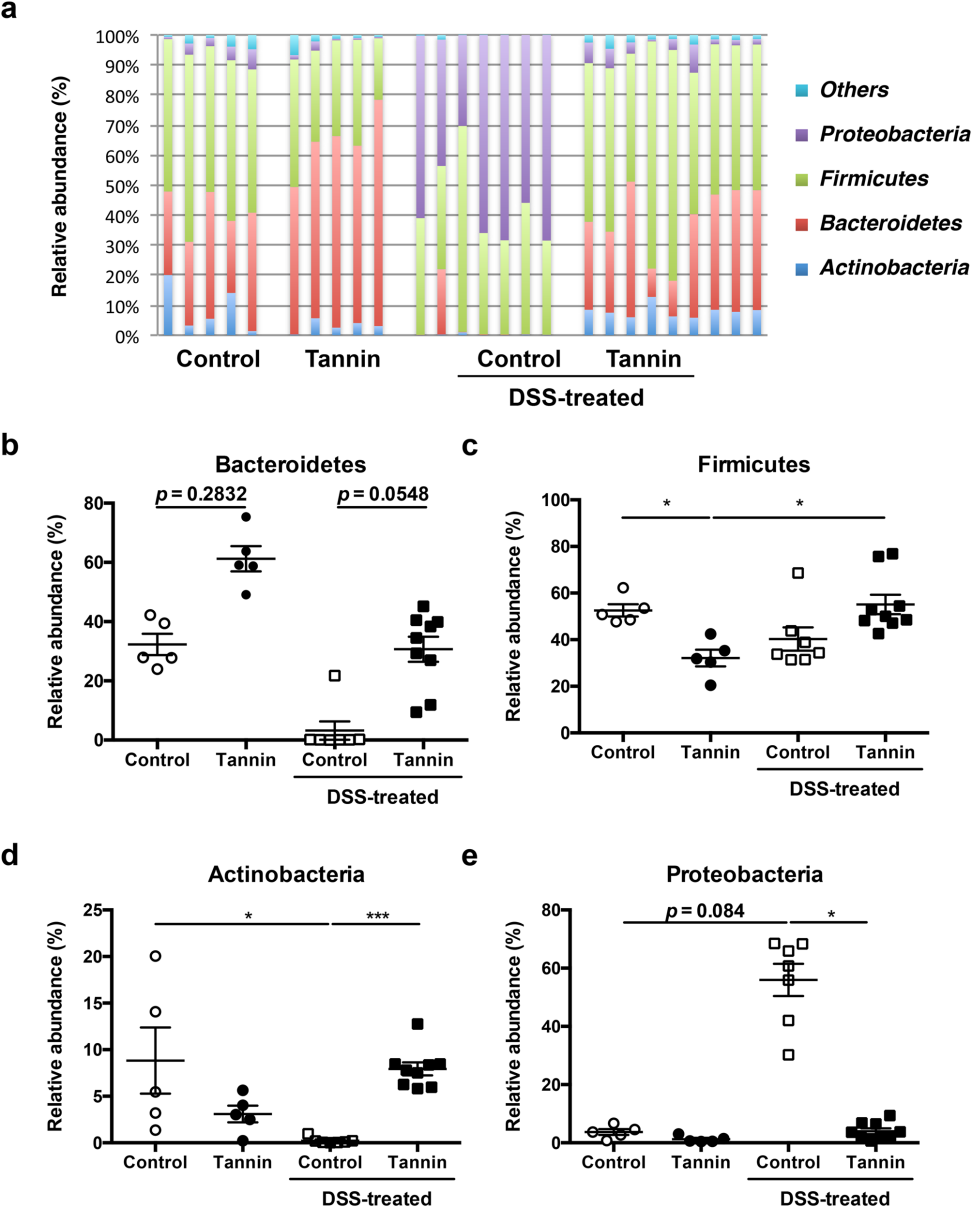
Dietary supplementation of persimmon-derived tannin alters the proportion of bacteria at the phylum level in colitis-induced mice. (**a**) Relative abundances of major phyla of faecal bacteria in untreated and DSS-treated mice fed the control or tannin-supplemented diet were analysed by 16S ribosomal RNA gene sequencing and shown in individual histograms. (**b**–**e**) Summarised data for the relative abundance of each phylum, Bacteroidetes (**b**), Firmicutes (**c**), Actinobacteria (**d**), and Proteobacteria (**e**) are shown as the mean ± SEM with individual plots (n = 5–9). *p < 0.05, ***p < 0.001.

Next, we analysed the microbiota composition at the phylum level. More than 90% of the microbiota consisted of four phyla, Actinobacteria, Bacteroidetes, Firmicutes, and Proteobacteria, which is similar to human gut microbiota (Fig. 5a). The relative abundance of Bacteroidetes tended to be higher in untreated mice fed the tannin diet compared with those fed the control diet (Fig. 5b). Conversely, the relative abundance of Firmicutes in the tannin diet group was significantly lower (Fig. 5c). The abundances of Actinobacteria and Proteobacteria, which were rarely observed compared with the other two major phyla, were comparable between control and tannin diet groups (Fig. 5d,e). Treatment with DSS in the control diet group caused a marked increase of Proteobacteria with almost complete loss of Bacteroidetes and Actinobacteria. Notably, the expansion of Proteobacteria and decrease of Actinobacteria induced by DSS treatment was suppressed in the tannin diet group. These results suggest that persimmon-derived tannin suppresses the alteration of the intestinal bacterial composition induced by DSS treatment.

**Figure 6:**
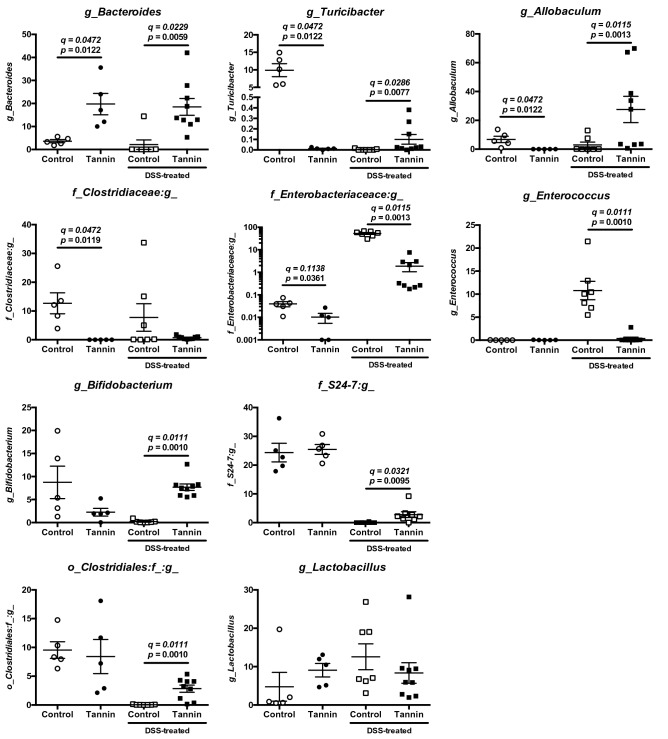
Dietary supplementation of persimmon-derived tannin suppresses expansion of potentially pathogenic bacteria in DSS-induced colitis. Relative abundances of 10 genera that were most contributed to dissimilarity of faecal microbiota in the untreated and DSS-treated mice fed the control or tannin-supplemented diet were shown as the mean ± SEM with individual plots (n = 5–9). Q-value is an FDR-adjusted *p* value.

To investigate which kinds of bacteria were regulated by persimmon-derived tannin, we assessed the bacterial genera that contributed to dissimilarity by Jaccard and Bray–Curtis distances, which corresponded to the indices of qualitative and quantitative dissimilarity, respectively (Supplementary Figure S3). The 10 genera that were most contributed to dissimilarity were consistent between Jaccard and Bray–Curtis distances. The relative abundances of these genera in each group were shown in Fig. 6. The *Bacteroides* was significantly increased in the tannin diet group and maintained during DSS-induced colitis. On the other hands, *Turicibacter, Allobaculum* and *Clostridiaceae* were decreased in tannin diet group. The *Enterobacteriaceae* and *Enterococcus*, which belong to phyla Proteobacteria and Firmicutes, respectively, were dramatically enriched in colitis-induced mice fed the control diet. Expansion of *Enterobacteriaceae* by DSS treatment was also observed in the tannin diet group, but the relative abundance of this bacterium was significantly lower in the tannin diet group compared with the control diet group. The increase of *Enterococcus* induced by DSS treatment was not observed in the tannin diet group. Interestingly, the *Bifidobacterium*, *Turicibacter, Allobaculum*, *S24-7* and unidentified genus of *Clostridiales* were increased in the colitis-induced mice fed tannin diet. Therefore, these alterations in microbiota by persimmon-derived tannin might contribute to ameliorating DSS-induced colitis.

## Discussion

Inflammation is a major factor for the progression of numerous chronic diseases and disorders including diabetes, cardiovascular diseases, cancer, arthritis, obesity, autoimmune diseases, and IBD^20,21^. Natural products play a significant role in human health in relation to the prevention and treatment of inflammatory conditions^22^. Consequently, natural effective anti-inflammatory compounds as safe and effective therapeutic agents have gained increasing attention. Persimmon-derived tannin is a condensed polymeric tannin consisting of flavan-3-ols such as catechin groups. In Japan, persimmon-derived tannin is processed into kaki-shibu and used for waterproofing and dyeing leather and cloth. Recently, it was demonstrated that persimmon-derived tannin has a beneficial anti-inflammatory effect in various disease models^23–25^. We therefore hypothesised that persimmon-derived tannin may have an anti-inflammatory effect against IBD and thus prevent the inflammation of IBD.

Here, we first demonstrated that the pathogenesis of UC model mice fed persimmon-derived tannin was improved compared with the control diet group. Gene expression of an inflammatory cytokine (IL-1β) and chemokine (CXCL1) was decreased significantly in the tannin diet group. Macrophages are critical for the development of inflammatory reactions in IBD because they produce various pre-inflammatory mediators. Macrophages sense conserved molecular patterns on microbes (pathogen-associated molecular patterns) via germ-line encoded pattern recognition receptors such as TLRs^26^. We therefore investigated whether persimmon-derived tannin has an anti-inflammatory effect under a CpG-stimulated inflammatory condition. CpG, a TLR9 ligand, mimics the immune stimulatory effect of bacterial DNA, which promotes proinflammatory cytokine induction and facilitates maturation/activation of macrophages. Gene expression of proinflammatory cytokines (IL-1β, IL-6, and TNF-α) and iNOS induced by CpG stimulation was significantly inhibited by treatment with the hydrolysate of tannin, which might facilitate regulating excessive innate inflammatory responses.

Gut-associated lymphoid tissues, especially various subsets of effector CD4^+^ helper T cells, also contribute to the extent of inflammation in IBD^27^. Th1 cells, which produce IFN-γ, accumulate in the intestinal tract of CD patients^28^. Animal colitis models of IFN-γ-deficient mice show loss of IFN-γ, which correlates with a reduction of inflammation and tissue damage^29^. Th17 cells, which produce IL-17 and IL-22, have also been implicated in the induction and promotion of UC pathogenesis, although IL-17 has both protective and pathological functions^2^. Chen et al*.* showed that proanthocyanidins, which are condensed tannins, attenuate allergic contact dermatitis by directly inhibiting T cell activation and Th1/Th17 cell responses^30^. Our data revealed that the proportions of Th1 and Th17 cells in the mesenteric lymph node and lamina propria were not significantly reduced in the persimmon-derived tannin diet group. IFN-γ and IL-17A mRNA levels tended to decrease in the colon of the tannin diet group, but not significantly, which suggested that persimmon-derived tannin mainly affected innate immunity or the microbiota rather than the development and/or functions of effector T cells in the colitis model.

Intestinal microbiota plays an important role in intestinal homeostasis and imbalance of the commensal bacterial community, dysbiosis, contributes to the pathogenesis of IBD. One of the major patterns of dysbiosis associated with IBD patients is a relative increase of bacterial species belonging to *Enterobacteriaceae*^7,31^. Although the microbiota of healthy individuals is mainly dominated by strict anaerobic bacteria belonging to Bacteroidetes and Firmicutes, facultative anaerobic *Enterobacteriaceae* can grow in not only an anaerobic, but also an aerobic environment, which is thought to be caused by inflammation^31^. Similar to IBD patients, overgrowth of *Enterobacteriaceae* has been observed in various kinds of colitis models such as genetic ablation of IL-10, pathogen-induced inflammation, and chemically induced inflammation by DSS^32^. In this study, robust expansion of *Enterobacteriaceae* by DSS treatment was also observed in the control diet group. This expansion was markedly suppressed in the tannin diet group. It is well known that tannin has an antimicrobial activity and several reports have shown that condensed tannin inhibits the growth of *Escherichia coli* that belongs to *Enterobacteriaceae*^33,34^*.* In addition, we showed that persimmon-derived tannin and its hydrolysate suppressed induction of iNOS mRNA expression in the inflamed colon and stimulated macrophages. Nitric oxides, which increase during colon inflammation in UC animal models and patients, enhance the growth of *E. coli* by nitrate respiration^35,36^. Although these possible mechanisms by persimmon-derived tannin may contribute to the suppression of *Enterobacteriaceae* expansion and colon inflammation, further experiments using gnotobiotic colitis models mono-associated with various strains of *Enterobacteriaeae* were required for elucidation of mechanisms^37^.

Most polyphenols in a diet (more than 90%) reach the large intestines and ferment into low molecular weight phenolic metabolites, that have antioxidant, antimicrobial, and anti-inflammatory activities by being absorbed in the body or remaining in the lumen^15^. It has been reported that dietary supplementation of epigallocatechin gallate, one of the components of tannin, increases the relative abundance of *Bacteroides* in rats^38^. In the present study, supplementation of persimmon-derived tannin increased the abundance of *Bacteroides* in mice without DSS administration. Higher abundance of *Bacteroides* was retained in DSS-treated mice fed persimmon-derived tannin. *Bacteroides fragilis*, a symbiont human commensal bacterium, prevents colitis caused by potentially pathogenic *Helicobacter hepaticus* through production of polysaccharide A^39^. Additionally, some species of *Bacteroides* secrete large proteins with an antimicrobial activity^40^. In line with animal studies, it has been reported that the abundance of *Bacteroides* is reduced in mucosal samples of IBD patients^41^. These observations suggest that increased *Bacteroides* by dietary supplementation of tannin suppresses or competes with the expansion of potentially pathogenic bacteria such as *Enterobacteriaceae* and *Enterococcus*, which ameliorate colitis. Further investigation is required to determine whether expansion of *Bacteroidetes* by dietary supplementation of tannin directly influences expansion of pathogenic bacteria and the pathogenesis of colitis.

Supplementation of persimmon-derived tannin also increased the abundance of *Bifidobacterium* in DSS-treated mice, although increase of *Bifidobacterium* was not observed in the tannin-supplemented mice without DSS administration. *Bifidobacterium* is one of the major components of human intestinal microbiota, and well characterized as probiotics candidates for allergic diseases and IBDs^42,43^. Some strains of *Bifidobacterium*, such as *Bifidobacterium bifidum*, *breve* and *longum*, have the immunomodulatory property and intestinal epithelial barrier function^44^. Numerous studies reported that oral administration of probiotics including such strains reduced the course of disease in animal colitis models as well as IBD patients^43^. Since it is not clear that contribution of increased *Bifidobacterium* in tannin-supplemented mice to the amelioration of colitis, the functionality of this bacterium should be assessed in future investigation. In addition, further studies focusing on the increase of *Bifidobacterium* during the colitis by tannin supplementation are expected to provide insight into the maintenance of gut homeostasis by probiotics.

In this study, we clearly demonstrated that dietary supplementation of persimmon-derived tannin ameliorates the pathogenesis of DSS-induced colitis by suppression of the inflammatory response and alteration of the microbiota composition, especially suppression of *Enterobacteriaceae* expansion. Persimmon-derived tannin is a highly safe product extracted from a natural plant and might be a valuable candidate for IBD therapy.

## Methods

### Persimmon-derived tannin and tannin-supplemented diet

Persimmon soluble tannin powder was provided by Ishii Bussan Inc. (Nara, Japan) and stored at − 20 °C until use. The procedure for tannin extraction has been described previously^16^. Briefly, immature persimmon fruits harvested in Nara, Japan, in 2011 were treated with 0.2% ethanol for 5 days, crushed into small pieces, and then placed in water for 2 days. After discarding the supernatant, tannin was extracted from the residue by heating in water at 120 °C for 30 min. The extracted tannin was filtered, evaporated *in vacuo*, and dried at 160 °C to obtain soluble tannin powder. A batch of soluble tannin powder contained 75.5% condensed tannin measured as epigallocatechin gallate equivalent on the basis of the Folin-Ciocalter method^45^. The persimmon tannin-supplemented diet was prepared by adding 2% soluble tannin powder substituted for an equal amount of cellulose to the AIN-93G-modified basal diet (CLEA Japan Inc, Tokyo, Japan). A detailed composition of the diet has been described previously^16^. For in vitro experiments, 100 mg of soluble tannin powder was dissolved in 5 ml of a 1.2 M hydrochloric acid-50% methanol solution, heated at 90 °C for 3 h, and then diluted to 10 ml using the same solution.

### Animals and colitis model

BALB/c mice (7-week-old females) purchased from Japan SLC (Hamamatsu, Shizuoka, Japan) were maintained under specific pathogen-free conditions at the Animal Research Center of Nara Medial University. DSS (36,000–50,000 molecular weight, colitis grade) was purchased from MP Biomedicals (Santa Ana, CA). Mice were ad libitum fed the control diet (AIN93-G) or tannin-supplemented diet for 4 weeks and then colitis was induced by administration of 3% DSS in drinking water ad libitum for 5 days, followed by water for 2 days as shown in Fig. 1a (total n = 20 in each group). Faeces were collected from these mice at day 0 (untreated) and day 7 (DSS-treated), immediately frozen by dry ice, and then stored at -80 °C until analysis. The mice were monitored daily and DAI was calculated from weight loss (0 =  < 1%, 1 = 1–4.99%, 2 = 5–10%, and 3 =  > 10%), stool consistency (0 = normally formed pellets, 1 = soft pellets not adhering to the anus, 2 = very soft pellets adhering to the anus, and 3 = liquid stool on long streams), and rectal bleeding (0 = none, 1 = small spots of blood in stool and a dry anal region, 2 = Large spots of blood in stool and blood appears through the anal orifice, and 3 = deep red stool and blood spreads largely around the anus)^46^. The animal experimental protocols were approved by The Animal Care and Use Committee at Nara Medical University (Approval No. 11570). All procedures were carried out in accordance with the Policy on the Care and Use of Laboratory Animals, Nara Medical University and Animal Research: Reporting of In Vivo Experiments (ARRIVE) guideline.

### Histological analysis

Large intestines were surgically excised, measured for length, longitudinally cut, and fixed with 4% paraformaldehyde. After embedding in paraffin, sections were sliced to a thickness of 1–2 μm and stained with haematoxylin and eosin. The histological score was calculated by the severity of inflammation (0 = no inflammation, 1 = mild, 2 = moderate, and 3 = severe), thickness of inflammatory involvement (0 = no inflammation, 1 = mucosa, 2 = mucosa plus submucosa, and 3 = transmural), epithelial damage (0 = intact epithelium, 1 = disruption of architectural structure, 2 = erosion, and 3 = ulceration), and the extent of lesions (0 = no lesions, 1 = punctuate, 2 = multifocal, and 3 = diffuse)^47^.

### Quantitative RT-PCR

Total RNA was purified from the distal colon region using NucleoSpin RNA (Macherey–Nagel) and then reverse transcribed to cDNA with Superscript IV (Thermo Fisher Scientific, Waltham, CA). Quantitative PCR was performed with TaqMan gene expression assays on a StepOne (Thermo Fisher Scientific) using default cycling conditions. The following TaqMan gene expression assays were used: *Gapdh* (Mm99999915), *Il1b* (Mm00434228), *Il6* (Mm00446190), *Tnf* (Mm00443258), *Cxcl1* (Mm04207460), *Ccl2* (Mm00441242), *Nos2* (Mm00440502), *Muc2* (Mm01276696), *Ifng* (Mm01168134), *Il17a* (Mm00439618), and *Tbp* (Mm01277042). Gene expression was analysed by the relative ΔΔCT method and normalised to *Gapdh* or *Tbp* expression.

### Flow cytometric analysis

Lamina propria lymphocytes in the colon were isolated using a Lamina propria Dissociation Kit with a gentleMACS dissociator (Milteny Biotec GmbH, Bergisch Gladbach, Germany) in accordance with the manufacturer’s instruction. Briefly, colon pieces were preincubated with Hank’s balanced salt solution containing 1 mM dithiothreitol, 5 mM EDTA, and 0.1% bovine serum albumin, and then digested and minced with the enzymes in the Lamina propria Dissociation Kit with the gentleMACS dissociator to obtain a single cell suspension. Cells from the lamina propria and mesenteric lymph nodes were stained with Zombie-Aqua or Zombie-NIR (Biolegend, San Diego, CA) to exclude dead cells from the analysis. After blocking Fc receptors with an anti-CD16/32 monoclonal antibody (mAb) (eBioscience, San Diego, CA), cells were stained with mAbs against surface antigens. For intracellular antigen staining, cells were fixed and permeabilized with a Mouse Foxp3 Buffer Set (BD Biosciences, San Jose, CA) or Fixation/Permeabilization kit (eBioscience) and then stained with mAbs against intracellular antigens. For intracellular cytokine staining, cells were stimulated with phorbol 12-myristate 13-acetate (50 ng/ml, Sigma-Aldrich, St. Louis, MO) and ionomycin (500 ng/ml, Sigma-Aldrich) in the presence of Goldistop (BD Biosciences) for 4 h. After stimulation, cells were stained for surface and intracellular antigens. The following mAbs were used: BV421-labelled anti-CD45 (30-F11), FITC-labelled anti-CD4 (GK1.5), FITC-labelled anti-IFN-γ (11–7311), PE-labelled anti-CD25 (PC61), PE-labelled anti-IL-17A (TC11-18H10), PE-Cy7-labelled anti-CD3 (145-2C11), APC-labelled anti-CD4 (GK1.5), Alexa Fluor 647-labelled anti-Foxp3 (MF23), and APC-Cy7-labelled anti-CD8a (53–6.7). Stained cells were analysed using a MACSQunat Analyzer (Miltenyi Biotec). Data were analysed by FlowJo software (Tree Star, Ashland, OR).

### Generation and stimulation of BMMCs

Generation of macrophages from bone marrow cells has been described elsewhere^16^. Briefly, bone marrow cells were isolated from the tibia and femur of a mouse and then cultured with 10 ng/ml M-CSF (PeproTech, Cranbury, NJ) for 6 days to differentiate into macrophages. BMMCs were pretreated with various concentrations of tannin hydrolysate (0–100 μg/ml) for 2 h. After washing with PBS, the cells were stimulated with TLR9 ligand CpG ODN1826 (1 nM, Invivogen, San Diego, CA) for 4 h. After stimulation, the cells were collected for analysis of mRNAs by quantitative RT-PCR.

### Microbiota analysis

Total 29 faecal samples (untreated mice fed control or tannin diet, n = 5 each; DSS-treated mice fed control or tannin diet, n = 9 or 10, respectively) were used in the following procedure. Extraction of bacterial DNA, sequencing, and software analysis have been described previously^48^. Briefly, faecal samples (20 mg) from the mice were crashed in saturated phenol by glass beads (300 mg, 0.1 mm in diameter) and a Multi-Beads Shocker (Yasui Kikai Co., Osaka, Japan), and then bacterial DNA was extracted by phenol/chloroform and isopropanol precipitation. The V3–V4 region of bacterial 16S ribosomal RNA gene was amplified by PCR using the following primer sets: Tru357F (5′-CGCTCTTCCGATCTCTGTACGGRAGGCAGCAG-3′) and Tru806R (5′-CGCTCTTCCGATCTGACGGACTACHVGGGTWTCTAAT-3′). Total 26 in 29 samples were succeeded PCR amplification (untreated mice fed control or tannin diet, n = 5 each; DSS-treated mice fed control or tannin diet, n = 7 or 9, respectively). The PCR product was further amplified using the following barcoded primers adapted for Illumina MiSeq: Fwd 5′-AATGATACGGCGACCACCGAGATCTACACXXXXXXXXACACTCTTTCCCTACACGACGCTCTTCCGATCTCTG-3′ and Rev 5′-CAAGCAGAAGACGGCATACGAGATXXXXXXXXGTGACTGGAGTTCAGACGTGTGCTCTTCCGATCTGAC-3′, where X represents a barcode base. After purification by a QIAquick PCR Purification kit (Qiagen, Valencia, CA), equal amounts of amplicons were pooled and purified with a GeneRead Size Selection Kit (Qiagen). The pooled libraries were sequenced by the Illumina Miseq system with a MiSeq Reagent Kit v3 (Illumina Inc, San Diego, SA). Sequence data were analysed using the QIIME software package version 1.8.0 (http://qiime.org/) after removal of phiX reads from raw paired-end reads. Potential chimeric sequences were removed by UCHIME and then OTUs were assigned by open-reference OTU picking with a 97% threshold of pairwise identity. A total of 233,345 high-quality paired sequences were obtained from the 26 samples, with 8,975 ± 1,737 (average ± standard deviation) reads per sample. All sequences were used for the taxonomical classification performed using the Greengenes reference database (http://greengenes.secondgenome.com /downloads/database/13_5). Equal numbers of sequence (6470 reads) were used for the analysis for Alpha- and Beta-diversity. Alpha-diversity indices (number of OTUs, Chao1, PD whole tree, and Shannon index) and Beta-diversity indices (weighted and unweighted UniFrac distances) were calculated by QIIME software version 1.8.0. Beta-diversity indices (Jaccard and Bray–Curtis distances) were calculated by R 'vegan' package (version 2.5–7).

### Statistical analysis

Data are presented as the mean ± SEM and are representative of at least two independent experiments. Statistical analysis of weight change, colon length, and in vitro gene expression was performed by the Student’s *t*-test using GraphPad Prism 6 (GraphPad Software, San Diego, CA). *P* values of < 0.05 were considered statistically significant. Statistical analysis of the DAI score, histological score, and gene expression in the colon was performed by the Mann–Whitney *U*-test. Differences in microbiota at the phylum level and α-diversity indices were analysed by the one-way ANOVA with Kruskal–Wallis test. Intergroup differences were analysed using the Mann–Whitney *U*-test and adjusted by the false discovery rate (FDR) using the Benjamin and Hochberg method in Python version 3.8 (https://www.python.org/).

## Supplementary Information

Supplementary Figures.
