# Supplementary material for: Persimmon-derived tannin ameliorates the pathogenesis of ulcerative colitis in a murine model through inhibition of the inflammatory response and alteration of microbiota
Source: Sci Rep. 2021 Mar 31;11:7286. doi: 10.1038/s41598-021-86608-1 (PMC8012611; doi:10.1038/s41598-021-86608-1)
Supplement: Supplementary file 1 — Supplementary Figures. [file 41598_2021_86608_MOESM1_ESM.pptx]

## Slide 1
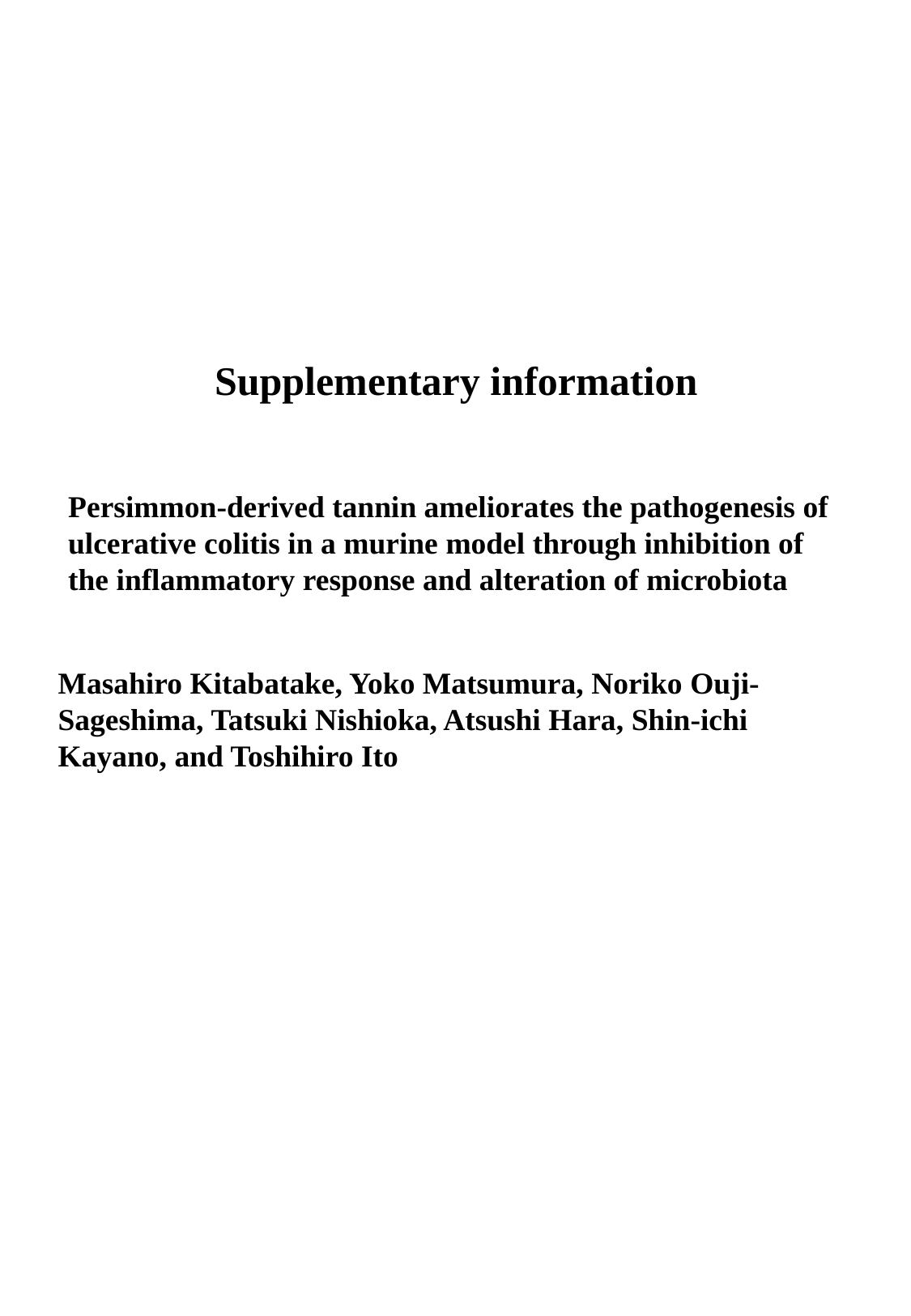

Supplementary information
Persimmon-derived tannin ameliorates the pathogenesis of ulcerative colitis in a murine model through inhibition of the inflammatory response and alteration of microbiota
Masahiro Kitabatake, Yoko Matsumura, Noriko Ouji-Sageshima, Tatsuki Nishioka, Atsushi Hara, Shin-ichi Kayano, and Toshihiro Ito

## Slide 2
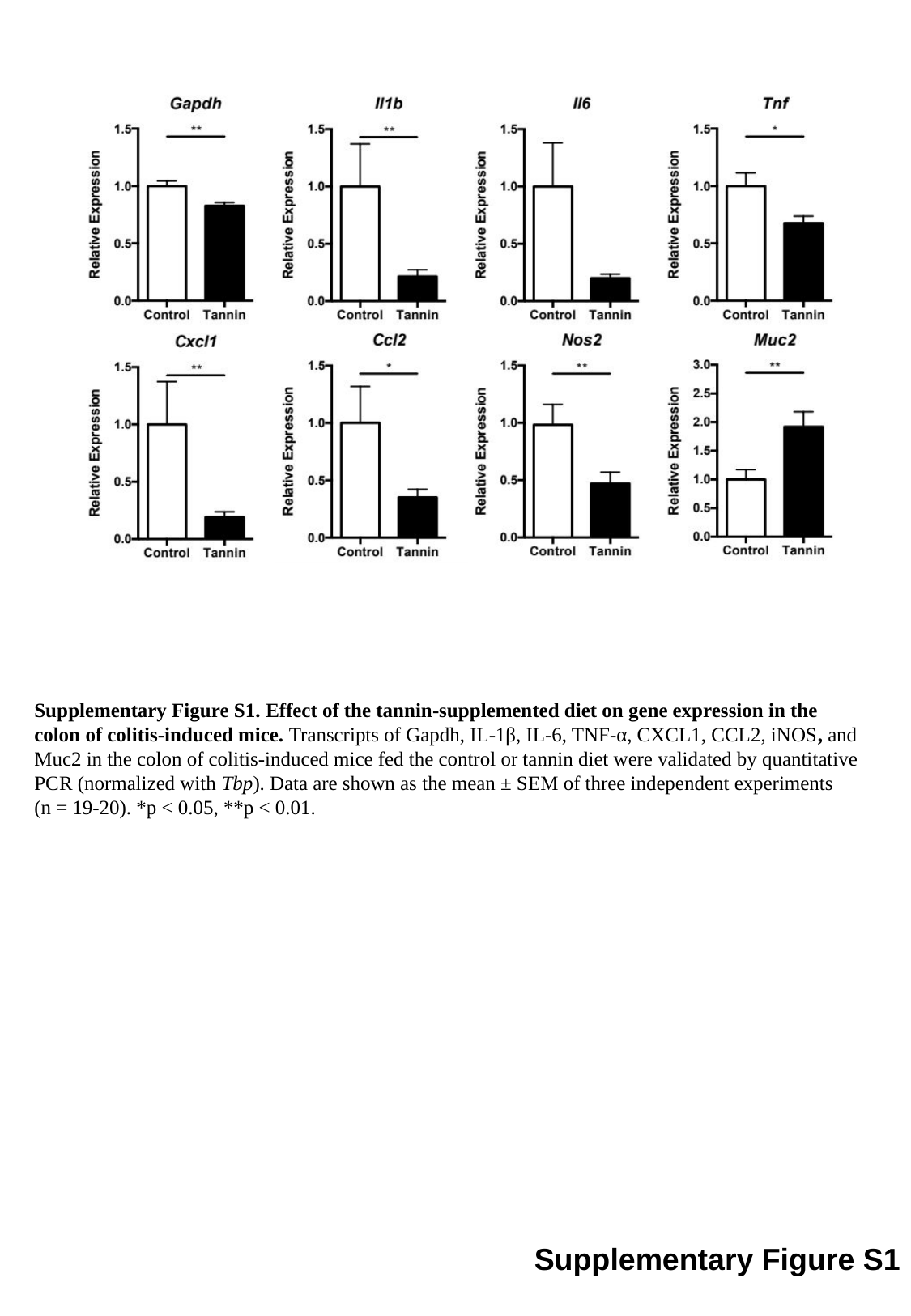

Supplementary Figure S1. Effect of the tannin-supplemented diet on gene expression in the colon of colitis-induced mice. Transcripts of Gapdh, IL-1β, IL-6, TNF-α, CXCL1, CCL2, iNOS, and Muc2 in the colon of colitis-induced mice fed the control or tannin diet were validated by quantitative PCR (normalized with Tbp). Data are shown as the mean ± SEM of three independent experiments
(n = 19-20). *p < 0.05, **p < 0.01.
Supplementary Figure S1

## Slide 3
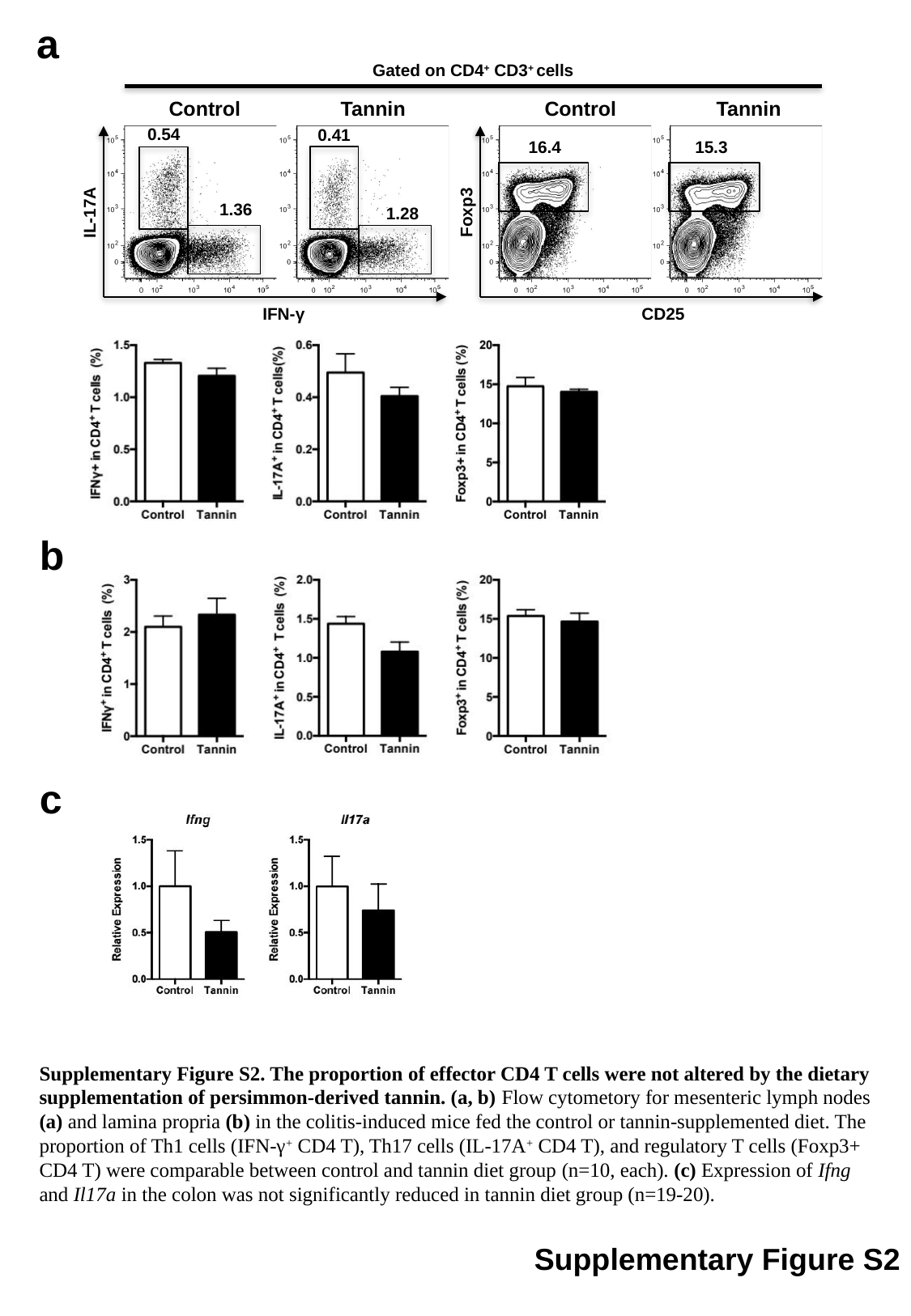

a
Gated on CD4+ CD3+ cells
Control
Tannin
Control
Tannin
0.54
0.41
16.4
15.3
1.36
IL-17A
Foxp3
1.28
IFN-γ
CD25
b
c
Supplementary Figure S2. The proportion of effector CD4 T cells were not altered by the dietary supplementation of persimmon-derived tannin. (a, b) Flow cytometory for mesenteric lymph nodes (a) and lamina propria (b) in the colitis-induced mice fed the control or tannin-supplemented diet. The proportion of Th1 cells (IFN-γ+ CD4 T), Th17 cells (IL-17A+ CD4 T), and regulatory T cells (Foxp3+ CD4 T) were comparable between control and tannin diet group (n=10, each). (c) Expression of Ifng and Il17a in the colon was not significantly reduced in tannin diet group (n=19-20).
Supplementary Figure S2

## Slide 4
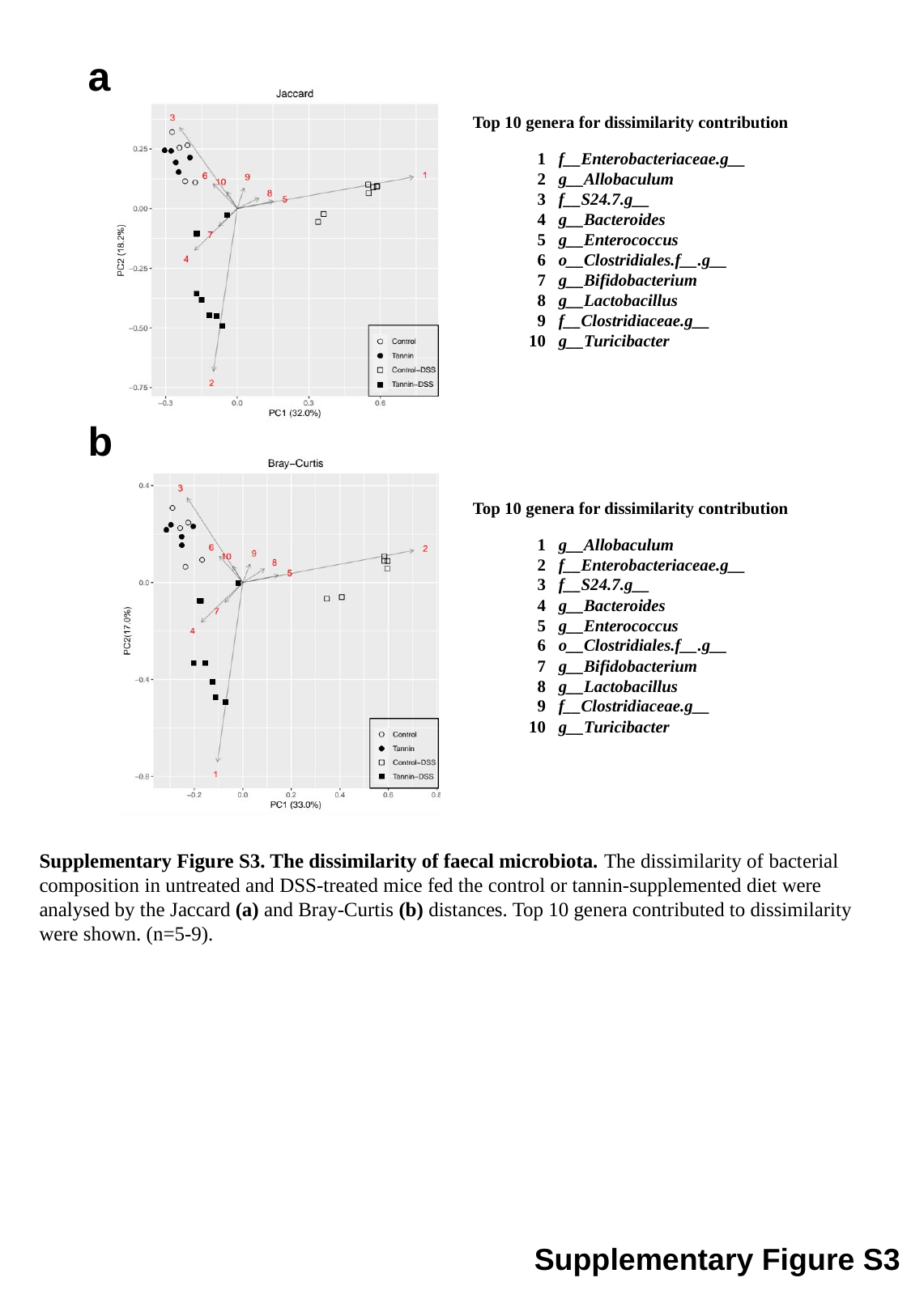

a
Top 10 genera for dissimilarity contribution
 1 f__Enterobacteriaceae.g__
 2 g__Allobaculum
 3 f__S24.7.g__
 4 g__Bacteroides
 5 g__Enterococcus
 6 o__Clostridiales.f__.g__
 7 g__Bifidobacterium
 8 g__Lactobacillus
 9 f__Clostridiaceae.g__
10 g__Turicibacter
b
Top 10 genera for dissimilarity contribution
 1 g__Allobaculum
 2 f__Enterobacteriaceae.g__
 3 f__S24.7.g__
 4 g__Bacteroides
 5 g__Enterococcus
 6 o__Clostridiales.f__.g__
 7 g__Bifidobacterium
 8 g__Lactobacillus
 9 f__Clostridiaceae.g__
10 g__Turicibacter
Supplementary Figure S3. The dissimilarity of faecal microbiota. The dissimilarity of bacterial composition in untreated and DSS-treated mice fed the control or tannin-supplemented diet were analysed by the Jaccard (a) and Bray-Curtis (b) distances. Top 10 genera contributed to dissimilarity were shown. (n=5-9).
Supplementary Figure S3
